# Latest advances and prospects in the pathogenesis, animal models, and vaccine research of severe fever with thrombocytopenia syndrome virus

**DOI:** 10.3389/fimmu.2025.1624290

**Published:** 2025-06-26

**Authors:** Chenghao Chen, Jiaxuan Li, Yumo Zhou, Yuwen Zhang, Renjin Huang, Yanjun Zhang, Jianhua Li, Keda Chen

**Affiliations:** ^1^ School of Medical Technology and Information Engineering, Zhejiang Chinese Medical University, Hangzhou, China; ^2^ Zhejiang Key Laboratory of Public Health Detection and Pathogenesis Research, Department of Microbiology, Zhejiang Provincial Center for Disease Control and Prevention, Hangzhou, China; ^3^ School of Basic Medical Sciences, Zhejiang Chinese Medical University, Hangzhou, China; ^4^ Key Laboratory of Artificial Organs and Computational Medicine of Zhejiang Province, Shulan International Medical College, Zhejiang Shuren University, Hangzhou, China; ^5^ School of Public Health, Hangzhou Medical College, Hangzhou, China; ^6^ School of Laboratory Medicine and Life Sciences, Wenzhou Medical University, Wenzhou, China

**Keywords:** animal models, vaccine, severe fever with thrombocytopenia syndrome virus, Gn, GC

## Abstract

Severe Fever with Thrombocytopenia Syndrome Virus (SFTSV), a tick-borne phlebovirus first identified in China, causes severe illness characterized by high fever, thrombocytopenia, leukopenia, and, in some cases, multi-organ failure and death. With mortality rates ranging from 5% to 30% in endemic regions, SFTSV has emerged as a significant public health threat across East Asia, including South Korea and Japan, with potential for broader outbreaks. This review synthesizes recent advances in SFTSV animal models and candidate vaccines, highlighting their contributions and limitations. Current animal models, including mice, ferrets, and non-human primates, partially replicate human disease but fail to fully recapitulate clinical manifestations, limiting their translational utility. Vaccine development has shown promise, with candidates such as mRNA, subunit, and viral vector vaccines demonstrating efficacy in preclinical studies, yet none have progressed to clinical trials. Key challenges include viral genetic diversity and immune evasion. Future research should focus on refining animal models to better mimic human pathology, developing broad-spectrum vaccines, and integrating virological and immunological insights to enhance prevention and treatment strategies for SFTSV.

## 1 Introduction

Severe Fever with Thrombocytopenia Syndrome Virus (SFTSV), officially classified as Dabie bandavirus (Family *Phenuiviridae*, Genus *Bandavirus*) by the International Committee on Taxonomy of Viruses (ICTV) in 2020, is a tick-borne phlebovirus first isolated in 2009 from a patient in Henan Province, China (1). Since then, cases have been documented in South Korea, Japan, Vietnam, Myanmar, Thailand, Pakistan, and many other countries and regions (2–6). Reports of human bites by Haemaphysalis longicornis ticks in the United States have raised concerns about the potential emergence of SFTSV as a public health threat beyond Asia (7). According to the latest classification by the International Committee on Taxonomy of Viruses (ICTV) in 2020, SFTSV belongs to the family Phenuiviridae and the genus Bandavirus, and it has been officially named Dabie bandavirus (DBV) (8). However, the term SFTSV remains more widely used in academic literature. Thus, this article continues to refer to the virus as SFTSV.

The primary route of human infection with SFTSV is through tick bites (9). The incubation period for the infection typically lasts 7–14 days, with a reported mortality rate ranging from 5% to 30% (10). SFTSV infected patients generally progress through three phases: the febrile phase, multiorgan dysfunction (MOD) phase, and recovery phase. The febrile phase lasts approximately 7 days and is characterized by fever, thrombocytopenia, leukopenia, lymphadenopathy, and gastrointestinal symptoms (such as nausea, vomiting, and diarrhea) (11). High viral loads detected during this stage are a key marker for the clinical diagnosis of SFTSV infection (12). The MOD phase develops rapidly between days 7 and 13 post-infection, with elevated serum levels of alanine aminotransferase (ALT), aspartate aminotransferase (AST), alkaline phosphatase (ALP), and lactate dehydrogenase (LDH), along with prolonged activated partial thromboplastin time (aPTT). Clinical manifestations during this phase include hemorrhagic and neurological symptoms, disseminated intravascular coagulation (DIC), multi-organ failure, and persistent thrombocytopenia. These factors that are major determinants of mortality. The MOD phase is critical for determining the prognosis of SFTSV infected patients (13, 14). The recovery phase, which occurs between days 11 and 19, is marked by the resolution of clinical symptoms and the normalization of laboratory parameters. Multiple clinical studies have reported immunological parameters in clinical patients. Current evidence indicates that severe disease is primarily associated with a cytokine storm and elevated levels of pro-inflammatory molecules such as TNF-α, IP-10, and IL-6 (15). Furthermore, patients with severe disease exhibit lower counts of CD3^+^, CD4^+^, and CD8^+^ T cells compared to those with mild disease (16, 17). A reduction in the Th1/Th2 ratio and an increase in the Th17/Treg ratio are also related to disease severity (18). These changes suggest that an imbalance in cellular immunity is closely linked to progression to severe disease. In terms of humoral immunity, studies indicate that non-survivors lack NP or Gn specific IgG production, and that the distribution and function of B cell subsets are abnormal, with impaired antigen-presenting capacity (19). Additionally, age is a significant risk factor for SFTSV prognosis, with mortality predominantly observed in individuals aged 50 and above, and mortality rates increase with age (20). Due to the severity of SFTSV infection, the World Health Organization (WHO) has classified it as a priority pathogen requiring urgent attention (21).

Vaccines are the most effective public health intervention for preventing the spread of infectious diseases. Successful vaccination programs have eradicated many life-threatening diseases, such as smallpox and polio (22). The World Health Organization estimates that vaccines prevent 2 to 3 million deaths annually from diseases such as tetanus, pertussis, influenza, and measles (23). However, no approved vaccine for SFTSV is currently available. The SFTSV vaccines under development include live attenuated vaccines, inactivated vaccines, recombinant vector vaccines, subunit vaccines, DNA vaccines, and mRNA vaccines. All are still in the preclinical stage, with no candidate vaccines having progressed to clinical trials. Therefore, there is an urgent need to develop a broadly effective vaccine to address this potential public health threat.

## 2 SFTSV virology

### 2.1 Phylogeny

The family Bunyaviridae is the largest group of arboviruses, with over 300 identified species. Based on serological, morphological, and biochemical characteristics, this family is classified into five genera: Orthobunyavirus, Phlebovirus, Nairovirus, Hantavirus, and Tospovirus. Yu et al. conducted whole-genome sequencing of 12 SFTSV strains isolated from patients in China and identified the virus as an enveloped, segmented, negative sense spherical RNA virus belonging to the genus Phlebovirus (1). SFTSV was found to have close phylogenetic relationships with the Heartland virus (HRTV) discovered in the United States and the Malsoor virus identified in India (1, 24, 25).

### 2.2 Genomic structure and functions

The genome of SFTSV, like other viruses in the order Bunyavirales, comprises three RNA segments: L, M, and S. The L segment is negative sense RNA, consisting of 6,368 nucleotides, and encodes the RNA dependent RNA polymerase (RdRp), which is responsible for viral RNA replication and mRNA synthesis (26).

The M segment contains 3,378 nucleotides and encodes a glycoprotein precursor (Gp). This precursor is processed by host proteases into two subunits, Gn and Gc (27). Gn and Gc form the viral envelope and possess antigenic properties. During endocytosis, these glycoproteins mediate viral entry by binding to cellular receptors and inducing low-pH-dependent membrane fusion (28). Several factors, including non-muscle myosin heavy chain IIA (NMMHC-IIA), C-type lectin receptors such as dendritic cell-specific intercellular adhesion molecule-3-grabbing non-integrin (DC-SIGN), DC-SIGN-related molecules (DC-SIGNR), liver and lymph node sinusoidal endothelial cell C-type lectin (LSECtin), and UDP-glucose ceramide glucosyltransferase (UGCG), have been identified as key players in SFTSV entry (29–32). Gn/Gc proteins also serve as immunogenic targets, stimulating the production of specific neutralizing antibodies, thus providing valuable directions for future vaccine research (33). The efficacy of structural glycoprotein-based vaccines has been demonstrated in SARS-CoV-2, influenza virus, and Ebola virus (34–36).

The S segment consists of 1,744 nucleotides and employs an ambisense coding strategy to encode the nucleocapsid protein (NP) and non-structural protein (NSs) (37). NP is the most abundant protein in SFTSV particles and infected cells. It forms hexamers that encapsulate viral RNA (vRNA), creating ribonucleoprotein complexes (RNPs). This function protects the viral genome from degradation by host cell nucleases and innate immune responses (38, 39). Recent studies indicate that NP triggers mitophagy to degrade mitochondrial antiviral signaling proteins (MAVS), thereby blocking MAVS-mediated antiviral signaling and evading host immune defenses (40). NSs is an important virulence factor of SFTSV, which can inhibit the induction of type I interferons (41). Additionally, NSs targets the Tumor Progression Locus 2 (TPL2)-A20-binding NF-κB inhibitory factor 2 (ABIN2)-p105 complex, inducing interleukin-10 (IL-10) expression to enhance viral pathogenicity (42). At the same time, NSs also plays a crucial role in viral replication (43).

## 3 Epidemiology

### 3.1 Transmission

The transmission cycle and mechanisms of SFTSV in nature remain unclear. *Haemaphysalis longicornis* is the primary vector and an important reservoir of SFTSV (44). SFTSV RNA has also been detected in various tick species, such as the *Ixodes nipponensis*, *Dermacentor nuttalli*, and *Haemaphysalis flava*, in regions where SFTSV outbreaks occur (45–47). The parthenogenetic reproduction characteristic of the *Haemaphysalis longicornis* allows it to establish new populations more rapidly than sexual reproduction, accelerating the spread of the ticks (48).

Migratory birds have long been known to serve as long distance carriers of ticks that harbor various human pathogens, such as Crimean-Congo Hemorrhagic Fever Virus(CCHFV) and Tick-Borne Encephalitis Virus (TBEV) (49, 50). The distribution of *Haemaphysalis longicornis* aligns with the East Asia-Australia migratory bird routes, indicating that the spread of this tick species is likely linked to migratory birds (51).

**Figure 1:**
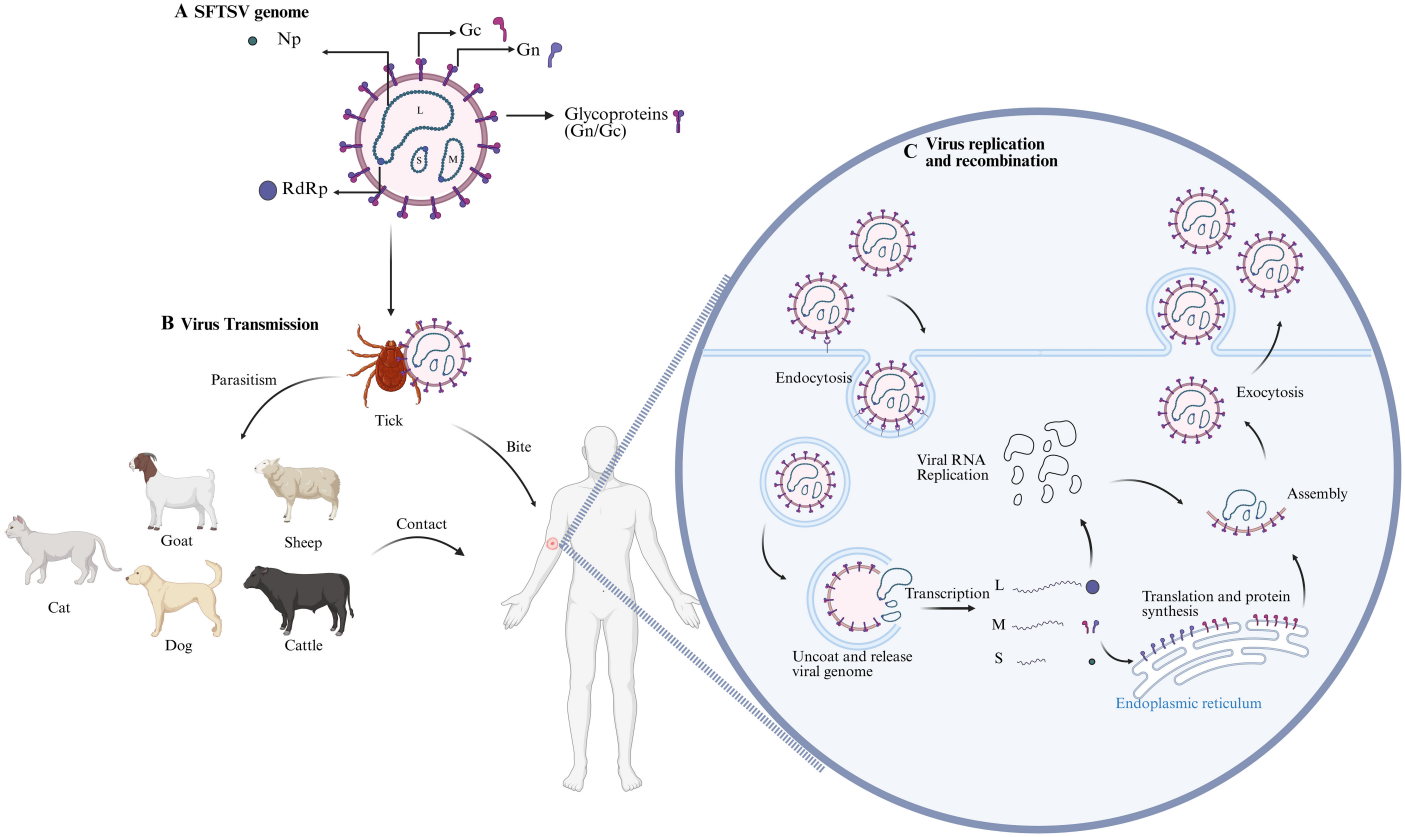
SFTSV Infection Mechanism Diagram. **(A)** Schematic of SFTSV structure, illustrating its enveloped nature and three RNA genome segments (L, M, S) encoding RNA-dependent RNA polymerase (RdRp), glycoproteins (Gn/Gc), and nucleocapsid (NP) and non-structural (NSs) proteins, respectively. **(B)** SFTSV is transmitted via Haemaphysalis longicornis tick bites to animal hosts (e.g., goats, cattle), with human infections occurring through direct contact or tick bites; human-to-human transmission is also possible. **(C)** The virus enters host cells via clathrin-mediated endocytosis, with Gn/Gc glycoproteins binding cellular receptors (e.g., DC-SIGN, NMMHC-IIA) and inducing low-pH-dependent membrane fusion. The viral genome is released and replicates in the cytoplasm. Glycoproteins are translated and modified in the host endoplasmic reticulum and Golgi apparatus. New viral particles assemble and are released via budding. The figure was created with BioRender.com.

Although the primary route of SFTSV infection in humans is through tick bites, human-to-human transmission of the virus has also been confirmed. Contact with the blood and bodily fluids of infected individuals can lead to virus transmission. Additionally, ticks may parasitize on a variety of wild animals and livestock, including birds and domestic animals, thereby infecting these animals and subsequently transmitting the virus to humans through close contact (52). SFTSV specific antibodies have been detected in a variety of animals, including goats, sheep, cattle, dogs, and cats, with the highest antibody positivity found in goats and cattle (53). However, these animals do not show obvious symptoms of infection and do not exhibit significant viremia, serving as reservoir hosts for SFTSV (**Figure 1**). The persistent infection cycle in animal hosts allows SFTSV to continue to spread in nature. Special protective measures should be taken for populations with close contact with these animals.

### 3.2 Genotype and recombination

Phylogenetic analysis of SFTSV strains has revealed six genotypes (A-F), with genotype B further subdivided into at least three distinct genotypes (B-1, B-2, and B-3) (54, 55). In mainland China, three of the six genotypes (F 43.6%, A 20.1%, D 15.4%) are predominant, while the B-2 subtype is prevalent in South Korea (36.1%) and Japan (86%) (54–56). The varying distribution of genotypes in SFTSV endemic regions leads to significant differences in mortality rates, with Japan (23%) and South Korea (27%) experiencing higher mortality rates compared to China (5.3%–16.2%) (57). Studies by Yun et al. have further demonstrated a close association between mortality rates, patient age, and SFTSV genotypes (55). Additionally, recombination plays an important role in the genetic diversity of segmented genome viruses. The segmented nature of the SFTSV genome leads to a high probability of recombination events. At least seven recombinant strains have been identified in China, while South Korea has reported at least nine recombinant genotypes (55, 58).

The genetic recombination phenomenon of SFTSV reveals its dynamic evolutionary characteristics in nature, presenting significant challenges for SFTSV diagnosis, epidemiological surveillance, treatment strategies, and vaccine development. Particularly in vaccine research, recombination significantly increases the genetic diversity of the virus, which may reduce the protective efficacy of neutralizing antibodies induced by vaccines against newly recombinant strains. Moreover, the high mutation rate and recombination properties of the virus further drive variation in antigenic epitopes, significantly enhancing the virus’s immune evasion capacity. Additionally, recombination may alter the pathogenicity and transmissibility of the virus, exacerbating immune protection discrepancies between different genotypes, leading to variable vaccine efficacy across regions. These complex factors pose key scientific challenges that need to be addressed with innovative research strategies and technological approaches for effective vaccine development.

Given the challenges posed by SFTSV’s genetic recombination, including heightened genetic diversity and immune evasion capabilities, vaccine development necessitates multidimensional strategies to enhance broad-spectrum efficacy and long-term protection: In terms of dynamic monitoring and evolutionary analysis: High-throughput sequencing can be employed to identify viral mutation hotspots, while the establishment of databases and bioinformatics analysis platforms enables the tracking of viral evolutionary dynamics across different regions and time periods, thereby providing the latest genetic information for vaccine design. Furthermore, by integrating structural biology techniques such as cryo-electron microscopy (cryo-EM) and X-ray crystallography to perform high-resolution structural analyses of key antigens (e.g., Gn and Gc), critical neutralizing antibody epitopes can be precisely mapped. The application of multi-target strategies and advanced technological platforms in vaccine design holds promise for overcoming the challenges posed by SFTSV gene recombination and mutation-induced immune evasion and diversity, ultimately leading to broader and more effective vaccine protection.

## 4 Animal models for SFTSV

Animal models are essential tools for studying the pathogenesis of viruses, developing vaccines, and exploring antiviral treatments. However, most animals do not exhibit fatal effects upon SFTSV infection, which presents a significant obstacle in the development of vaccines and antiviral therapies. It is crucial to thoroughly understand the pathogenesis of SFTSV in each model, taking into account the differences and similarities between animal models and human cases. Recent studies have identified several animal models highly susceptible to SFTSV infection. These models can mimic certain aspects of the pathogenesis observed in human SFTS, providing a basis for developing vaccines and antiviral drugs against SFTSV infection (**Table 1**).

**Table 1:** Animal models investigated for SFTSV.

Species	Animal strain	SFTSV strain	Lethality	Symptom of infection	Limitations	Advantages	Reference
Mouse	Adult C57BL/6 BALB/c, C3H, FVB	HB29; CB1/2014	Non-lethal	Viral RNA and histopathological changes were identified in the spleen, liver, and kidney,elevated AST, ALT, and BUN, megakaryocytes increased in both spleen and bone marrow	Mild symptoms of infection	Low cost, easily accessible, applicability	(59, 60)
	Newborn C57BL/6, BALB/c, Kunming, ICR (CD-1)	HYSV; YL-1	Lethal	Liver damage, high viral titers, neuronal necrosis(Kunming)	Immature immune system, pathological differences	High susceptibility, low cost, easily accessible	(61, 62)
	Mitomycin C-treated C57BL/6	HB29	Lethal	50% mortality	High cost,complexity, abnormal physiological state	Mimics immunosuppression, high susceptibility	(59)
	IFNAR Ab-treated C57BL/6	KH1	Lethal	Lesions in the liver, spleen, intestine, hematologic abnormalities			(63)
	IFNAR^−/−^ C57BL/6	YG-1; SPL 010	Lethal	Weight loss			(60, 64)
	IFNAR^−/−^ 129/Sv	YL-1	Lethal	Virus detected in blood, brain, heart, kidney, liver, lung, and spleen,100% mortality			(62)
	Aged BALB/c, C3H, C57BL/6, FVB	CB1/2014	Non-lethal	Mild weight loss	Short lifespan, mild infection symptoms	Aging model, well-established strains	(60, 65)
	STAT1^−/−^ C57BL/6	YG-1	Non-lethal	Mild symptoms	High cost, complexity	STAT2^−/−^ mice has high susceptibility	(66)
	STAT2^−/−^ C57BL/6	YG-1	Lethal	Weight loss, detectable virus in the brain, lungs, liver, spleen, kidney and intestines			
	HuPBL–NCG	E-JS-2013-24	Lethal	Multiple organ lesions,disruption of the vascular endothelial barrie,reduced PLT and WBC counts	High construction costs, long experimental period, B cell immune response requires further exploration	Human-like immune response, closer to human pathological features	(67)
	Human CD34 + HSC-transferred	WCH	Lethal	Multiple organ infection, reduced PLT and WBC counts,elevated ALT and AST levels			(68)
Rat	Adult wistar rat	HYSV	Non-lethal	100% mortality by i.c., 40% mortality by i.p.	Adult rats exhibit asymptomatic or mild symptoms, with a lack of studies on histopathological and hematological characteristics.	Newborn rat high susceptibility	(61)
	Newborn wistar rat	HYSV	Lethal	Not reported			
Hamster	Stat2−/− Syrian golden hamster	HB29	Lethal	Pathological lesions in liver and spleen;reduced PLT counts	High cost and limited research.	High susceptibility	(69)
Ferret	≤2 years ferret	CB1/2014	Non-lethal	Mild weight loss	High cost, limited model availability and insufficient research on the immune system	High susceptibility, clinical symptoms similar to severe human infections and high mortality rates	(65)
	≥4 years ferret	CB1/2014	Lethal	Elevated AST/ALT levels, high fever, virus replication in various tissues in the body			
Cat	Russian Blue American Shorthai	SPL010	Lethal	High fever, gastrointestinal symptoms, leukopenia, thrombocytopenia, and liver and kidney damage	High cost, age-independent susceptibility and limited research	Symptoms similar to severe human infections.	(70)
Non-human primates	Rhesus macaque	HB29	Non-lethal	Mild,fever, thrombocytopenia, leukopenia, and elevated AST/ALT levels	Mild infection symptoms, high research costs, strict ethical review requirements, and biological safety risks.	Highly similar to human genetics, physiology, and immune system.	(71)
	Cynomolgus macaque	SD4	Non-lethal	Slight decrease in platelet			(60)

This table summarizes animal models used for SFTSV research, including mice, rats, ferrets, cats, hamsters, and non-human primates. It lists animal strains, SFTSV strains, lethality (lethal or non-lethal), infection symptoms (e.g., thrombocytopenia, leukopenia, organ damage), advantages (e.g., accessibility, similarity to human pathology), limitations (e.g., high cost, immune system differences), and references. Models are categorized by species, with lethality and symptoms as key evaluation criteria, aiding vaccine and antiviral drug development.

### 4.1 Mice

#### 4.1.1 Immunocompetent mice

In an early study, Jin et al. intramuscularly injected 10^5^ TCID50 of the SFTSV HB29 strain into C57BL/6 mice, resulting in elevated levels of AST, ALT, and BUN (blood urea nitrogen), along with leukopenia and thrombocytopenia—pathological features similar to mild human infections. Pathological changes were observed in the liver, spleen, and kidneys, with viral RNA detected in all three organs, though viral replication was observed only in the spleen (59). Additionally, their research revealed that splenic macrophages were the target cells of infection. However, the C57BL/6 mouse model failed to progress into severe or fatal SFTSV infections. To induce fatal infections, researchers administered the immunosuppressant mitomycin C (59). Similarly, immunocompetent adult mouse strains such as BALB/c, C3H, FVB, and ICR (CD-1) showed no severe clinical manifestations upon SFTSV infection (59, 60).

#### 4.1.2 Age-dependent mice

Newborn mice, including C57BL/6, BALB/c, Kunming, and ICR (CD-1), are susceptible to SFTSV, exhibiting severe symptoms and high mortality rates (61, 62). Although newborn mice are highly sensitive to SFTSV and display significant pathological changes, their immune systems are not fully developed, and experimental operations are challenging. In comparison, aged mice (over 20 months old), such as BALB/c, C3H, C57BL/6, and FVB strains, show only slight weight loss (60, 65). Therefore, age-related mouse models may not be suitable for simulating the progression characteristics of SFTS in elderly human patients or for studying the age-specific pathogenesis of SFTSV.

#### 4.1.3 Immunocompromised mice

Immunodeficient gene knockout mice have also been employed to study SFTSV infections. These include α/β interferon receptor knockout (IFNAR^−/−^) mice (60, 62, 64), signal transducer and activator of transcription 2-deficient (STAT2^−/−^) mice, and mice treated with blocking anti-type I interferon (IFN)-α receptor antibody (IFNAR Ab-treated mice) (60, 62–64, 66). These models exhibit high susceptibility to SFTSV infection and lethal outcomes, with viral replication detected in multiple organs. Severe infection symptoms were observed, including significant weight loss, multi-organ pathological changes, severe leukopenia, and thrombocytopenia. Similar observations were made in the STAT2 knockout model of golden Syrian hamsters (69).

#### 4.1.4 Humanized mice

Two humanized mouse models constructed through immune system reconstitution have been reported as lethal models for SFTSV, providing a closer simulation of human infection compared to wild-type and immunocompromised mice. Li et al. transplanted highly purified (>90%) human CD34^+^ cells, isolated from umbilical cord blood, into mice via tail vein injection (68). SFTSV infection in these mice led to multi-organ involvement, reductions in platelet and white blood cell counts, and elevated ALT and AST levels, closely resembling the clinical characteristics of SFTSV patients. SFTSV infection in this humanized mouse model resulted in fatal outcomes. Xu et al. established the HuPBL-NCG mouse model by transplanting human peripheral blood mononuclear cells (PBMCs) into NCG mice (67). The human PBMCs transplanted into NCG mice provided early replication targets for the virus, while infected human monocytes transmitted the virus to mouse monocytes through an intercellular transmission mechanism, which is more efficient in viral infections. Their study also elucidated aspects of the pathogenesis of hemorrhagic syndrome, including apoptosis, membrane protein endocytosis, and cytokine stimulation. The HuPBL-NCG model mimics many pathological features of human SFTSV infection, including virus-induced histopathological changes, disruption of vascular endothelial barriers, thrombocytopenia, and leukopenia. While this humanized mouse model offers a valuable tool for investigating the pathological mechanisms of SFTSV infection, further exploration is required to study B cell immune responses in detail.

### 4.2 Rats

In the rat model, all newborn Wistar rats died after intracranial (i.c.) inoculation with 2×10^7^ copies, whereas 40% of the rats died following intraperitoneal (i.p.) injection with 3×10^7^ copies. However, adult rats all survived after vaccination (61). Further studies are needed to investigate the characteristics of the SFTSV rat model, as histopathological and hematological examinations have not been reported to date.

### 4.3 Ferrets

The ferret, with anatomical and physiological features similar to those of humans, has been widely used in the study of various infectious diseases (72, 73). Park et al. have demonstrated that SFTSV infection in ferrets is age-dependent (65). After SFTSV challenge, young ferrets (<2 years old) only exhibit mild symptoms such as weight loss and slight weight gain. While there are changes in AST/ALT levels, PLT and WBC counts, these quickly return to normal ranges. Additionally, viral RNA can be detected in the spleen, liver, kidneys, lungs, and serum. However, aged ferrets (>4 years old) are more susceptible to infection and exhibit symptoms similar to severe human cases of SFTSV infection. These include significant thrombocytopenia, leukopenia, elevated AST/ALT levels, high fever, and weight loss. In aged ferrets, systemic infection is also triggered, with viral RNA detected in various tissues, and death occurs within ten days post-infection, with a mortality rate of 93% (65).

Although the use of aged ferrets offers advantages in the study of lethal SFTSV infection models and vaccine development, several limitations exist. These include high costs, limited availability of effective aged ferret models, and a lack of resources for studying the immune system mechanisms in ferrets.

### 4.4 Cats

The detection of SFTSV RNA in cats was first reported in 2017 in serum samples from wild cats in South Korea (74). There have also been reports from Japan of veterinarians contracting SFTSV while treating infected cats (75). In a study by Park et al., four out of six cats that were infected with SFTSV via intravenous injection died within 10 days post-infection (70). Cats that died from SFTSV infection exhibited severe clinical manifestations, including high fever, gastrointestinal symptoms, leukopenia, thrombocytopenia, and liver and kidney damage, resembling the symptoms of severe SFTS in humans. However, there was no correlation between the age of the cats and the severity of the disease.

The clinical and histopathological features in cats, similar to those seen in severe human infections, suggest that cats could be a promising animal model for SFTSV research. However, there are significant limitations: compared to established rodent models commonly used in laboratories, the cost of using cats is higher, handling cats in laboratory settings is more challenging, and research on cat models remains limited. Furthermore, standardized experimental cat strains have yet to be developed.

### 4.5 Non-human primates

Non-human primates share genetic, physiological, and immune system similarities with humans, making their immune responses and disease progression closer to those observed in humans. They are ideal models for vaccine development and studies on infection mechanisms and have been widely used to investigate the infection and pathogenesis of bunyaviruses that cause hemorrhagic fever diseases (76–78). Jin et al. reported cases of rhesus monkeys infected with SFTSV, which exhibited symptoms such as fever, thrombocytopenia, leukopenia, and elevated levels of transaminases and myocardial enzymes in the blood. These symptoms resembled mild SFTS in humans, without causing severe illness or death (71). In another study involving cynomolgus monkeys, no significant clinical symptoms were observed following SFTSV infection, and viral RNA was not detected during the 14 day study period (60). While non-human primate models offer the advantage of physiological and immune system similarities to humans, their application in SFTSV vaccine development is limited due to strict ethical reviews, high research costs, biosafety risks, and the mild symptoms observed in SFTSV infections in these models.

### 4.6 Comparison and prospect of animal models

SFTSV vaccine and drug development primarily rely on aged ferrets (see Section 4.3) and immunocompromised mouse models (see Section 4.1.3). Aged ferrets and cats are among the few immunocompetent models showing lethality, though cat models exhibit transient inflammatory responses and low neutralizing antibody titers (70). Aged ferrets display sustained inflammation, closely mimicking severe human infections (65). Humanized mouse models (see Section 4.1.4) develop human-like pathology and immune responses, with longer survival, making them suitable for pathogenesis and antiviral studies (67). Rhesus macaque models infected with SFTSV exhibit robust Th1-type pro-inflammatory responses, widespread immune cell recruitment, and inflammatory mediator release, yet display mild clinical symptoms (71). In current vaccine research, immunocompetent mice are commonly used for preliminary evaluation of vaccine immunogenicity.

In general, future research should concentrate on refining animal models that show promise yet remain underutilized. For example, researchers can improve the reconstitution of the human immune system in humanized mice and employ genetic engineering techniques to optimize their microenvironment and genetic background. This approach may enable these models to more accurately simulate the immune response and vaccine-induced effects observed in human SFTSV infections. Moreover, for non-human primates that possess immune systems more akin to those of humans, adjusting infection conditions to intensify pathological manifestations or introducing immune regulatory strategies could help identify species or strains with heightened sensitivity. These efforts would contribute to the development of an animal model that more closely mirrors the severe clinical pathology seen in human SFTSV infections.

## 5 Advances in SFTSV vaccine research

**Figure 2:**
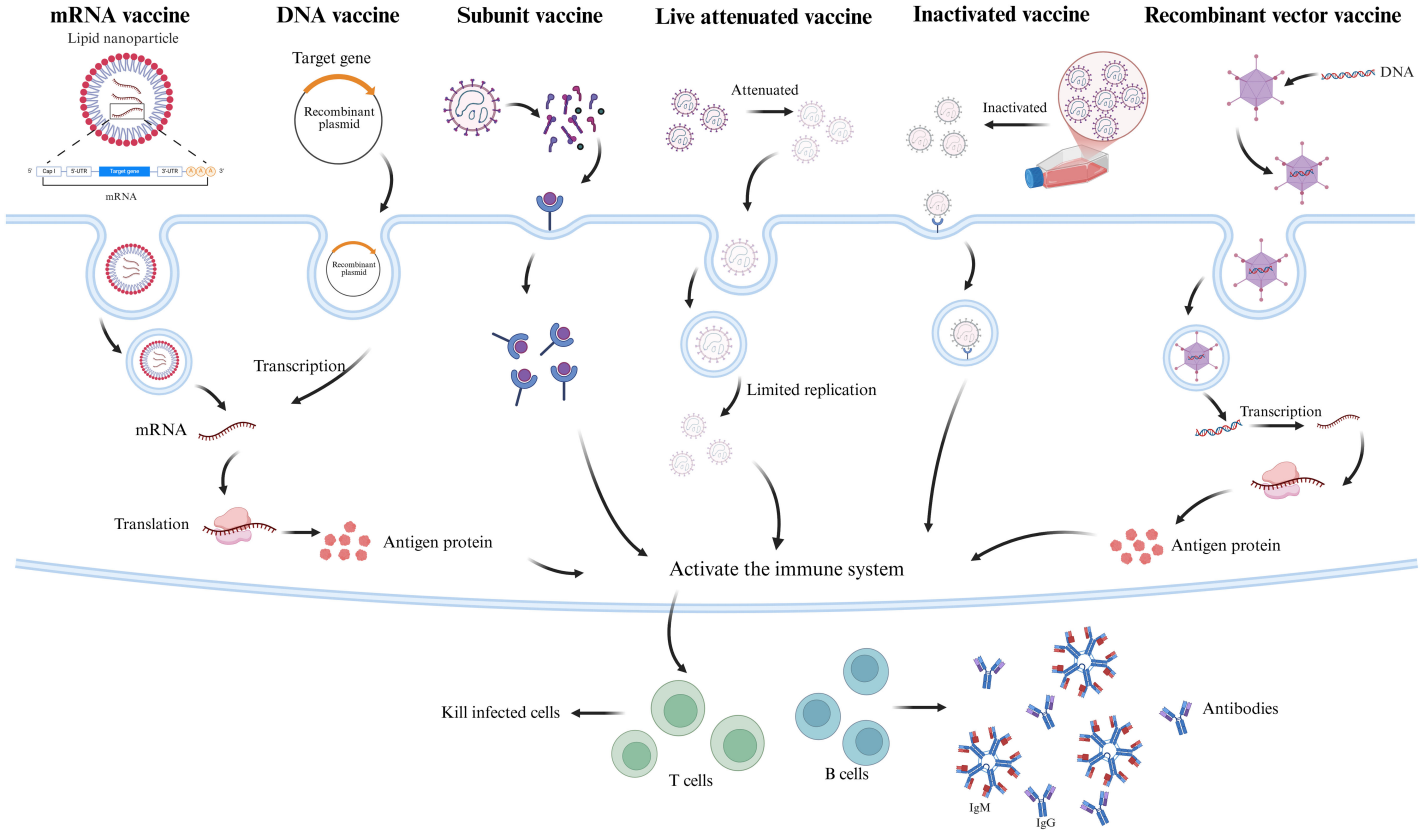
SFTSV Vaccine Mechanism of Action Diagram. This diagram illustrates the immune induction mechanisms of six SFTSV candidate vaccine types. mRNA vaccines, encapsulated in lipid nanoparticles (LNPs), deliver antigen-encoding mRNA (e.g., Gn, Gc) to the cytoplasm for translation into antigenic proteins. DNA vaccines, delivered via electroporation, are transcribed into mRNA and translated into antigens (e.g., Gn, Gc, NP). Subunit vaccines contain purified SFTSV antigens (e.g., Gn, Gc, NP). Inactivated vaccines use chemically inactivated (e.g., β-propiolactone) whole virus, retaining immunogenicity. Attenuated live vaccines, with reduced virulence via reverse genetics, mimic natural infection. Recombinant viral vector vaccines (e.g., VSV, adenovirus) express SFTSV antigen genes (e.g., Gn/Gc) in host cells. All vaccines activate B cells (producing neutralizing antibodies) and T cells (cytotoxic T cells), inducing specific humoral and cellular immunity against SFTSV infection. The figure was created with BioRender.com.

The current SFTSV candidate vaccines have achieved notable progress across various technological platforms (**Table 2**). To better illustrate their mechanisms of inducing immune responses, a simplified immune mechanism diagram is provided in **Figure 2**.

**Table 2:** SFTSV vaccines developed to date.

Type	Vaccine candidate	Antigen	Animal model	Post-challenge survival rate		Advantages	Limitations	References
				Immunization	Control			
Live attenuated vaccine	rHB2912aaNSs rHB29NSsP102A	SFTSV-NSs SFTSV-NSs	Ferret	100% 100%	0%	Cross-protective immunity, High genetic stability of rHB2912aaNSs	The genetic stability of rHB29NSsP102A during *in vivo* passaging remains to be studied	(79)
Inactivated vaccine	Whole Inactivated SFTSV	SFTSV-GP	C57/BL6 mice BALB/c mice	100%	100%	Safety, stability, mature preparation route	Low immunogenicity, requiring multiple vaccinations and adjuvant assistance	(80)
Recombinant vector vaccines	rVSV-SFTSV/AH12-GP	SFTSV-GP	C57/BL6 mice IFNAR^−/−^ mice	100%	0%	Cross-reactive protection, broad age applicability, multi-route immunization, no interference from pre-existing VSV-specific immunity	The long-term protective efficacy evaluations are needed	(81)
	m8-N m8-GPC m8-N+GPC	SFTSV-NP SFTSV-GP	IFNAR^−/−^ mice	100% 100% 100%	0%	Highly attenuated, production of SFTSV VLPs *in vitro*, no interference from pre-existing VAC-specific immunity	The role of cellular immunity in infection has not been fully clarified	(82)
	Ad5-G-Gn	RABV-G SFTSV-Gn	C57/BL6 mice BALB/c mice	100%	0%	High SFTSV VNA titer, high immunogenicity	Nonlethal murine model, further safety and protective efficacy evaluations are needed	(83)
	Ad5-Gn Ad5-Gc Ad5-Gn-Gc	SFTSV-Gn SFTSV-Gc	BALB/c mice IFNAR Ab-treated mice	100% 0% 12.5%	0%	High SFTSV VNA titer, high specificity and non-pathogenic	The Gc protein needs to be optimized to enhance its expression and immunogenicity in the vaccine	(84)
Protein subunit vaccines	(rSFTSV)/NSs	SFTSV-NSs	C57BL/6J mice	100%	100%	High titers of anti-NSs antibodies	No inhibition of viral replication nor accelerated viral clearance	(85)
	NP, Gn, Gc Gn + NP, Gc + NP Gn + Gc + NP	SFTSV-NP SFTSV-Gn SFTSV-Gc	C57/BL6 mice IFNAR^−/−^ mice	NP:66.7% Gn: 16.7% Gc: 0% Gn+NP:71.4% Gc+NP: 85.7% Gn + Gc + NP:57.1%	0%	Strong antigen-specific antibody and T cell responses	Immune response decline,no neutralizing activity,potential bias in immune response evaluation using IFNAR−/− mice model	(86)
	GnH-FT nanoparticles	SFTSV-Gn	Ferret	100%	0%	Safety, low dose high efficiency	High production costs and requires multiple immunizations	(87)
			BALB/c mice					
DNA vaccines	pVax1-Gn pVax1-Gc pVax1-N pVax1-NSs pVax1-RdRp	SFTSV-Gn SFTSV-Gc SFTSV-N SFTSV-NSs SFTSV-RdRp	Ferret BALB/c mice	100% 100% 100% 100% 100%	0%	The relative ease of development, broad immunity to multiple antigens	Single vaccination achieves only partial protection, requires multiple vaccinations	(88)
	pSFTSV⁃IL⁃12 pSFTSV	SFTSV-Gn/Gc SFTSV-NS/NP	IFNAR^−/−^ mice	100% 40%	0%	Easy to develop, broad immunity to multiple antigens	Insufficient immunogenicity, requires further optimization, possibility of side effects	(89)
mRNA vaccines	sGn-H mRNA LNPs sGn-H-FT mRNA LNPs	SFTSV-Gn	BALB/c mice IFNAR^−/−^ mice	100% 100%	0%	Strong immunogenicity, safety, low cost	Cross‐protective activity verification, further extended study period for antibody response and viral challenge	(90)
	GP-mRNA-LNPs	SFTSV-GP	BALB/c mice IFNAR^−/−^ mice	0.1μg: 90% 1μg: 100% 5μg: 100%	0%	Broad-spectrum protection, low dose high efficiency, long-term complete protection	Effectiveness needs to be evaluated in other animal models	(91)
	LNP-encapsulated mRNA-Gn vaccine	SFTSV-Gn	C57BL/6 mice	100%	0%	Safety, high efficiency, rapid production, low cost	The long-term protection and effectiveness in elderly individuals are unknown	(92)

This table summarizes current SFTSV candidate vaccines, including attenuated live, inactivated, recombinant viral vector, subunit, DNA, and mRNA vaccines. It lists vaccine candidate names, target antigens (e.g., Gn, Gc, NP), animal models (e.g., BALB/c mice, IFNAR−/− mice, ferrets), post-challenge survival rates (compared to controls), advantages (e.g., broad-spectrum protection, stability), limitations (e.g., need for multiple doses, unknown long-term efficacy), and references. Data aim to compare vaccine platform efficacy and development challenges.

### 5.1 Attenuated live vaccines

Attenuated live vaccines are a type of vaccine that weakens the pathogenicity of a pathogen to eliminate its virulence while retaining its immunogenicity. These vaccines can replicate to a limited extent within the host, simulating the natural infection process and inducing a strong and long-lasting immune response (93). Reverse genetics is an important tool for exploring the molecular biology and pathogenesis of RNA viruses, as well as for vaccine development. Currently, attenuation is achieved using reverse genetics methods (94).

Yu et al. generated two recombinant SFTSV strains from the HB29 strain via reverse genetics: rHB2912aaNSs (NSs ORF C-terminus truncated by 12 amino acids) and rHB29NSsP102A (proline-to-alanine mutation at NSs position 102). In aged ferrets, these strains exhibited attenuated phenotypes, inducing high neutralizing antibody titers within 14 days, sustained until day 58. Immunized ferrets achieved 100% survival post-lethal challenge (control: 0%). The rHB2912aaNSs strain maintained genetic stability after multiple passages, indicating low reversion risk (79).

### 5.2 Inactivated vaccines

Inactivated vaccines use chemical or physical methods to render pathogens non-infectious while preserving their immunogenicity, thereby eliciting an immune response in the host. Compared to live attenuated vaccines, inactivated vaccines are more stable and safer, making them a better option for immunocompromised individuals. However, their immunogenicity is relatively low, necessitating multiple doses and the use of adjuvants to enhance the immune response.

Li et al. isolated the SFTSV strain AH12 from patient blood samples and inactivated it by β-propiolactone (BPL). The whole virus particles were purified using ultrafiltration and ultracentrifugation techniques (80). The inactivated vaccine induced high levels of SFTSV specific IgG antibodies and neutralizing antibodies in BALB/c and C57/BL6 mice, with higher doses of the vaccine resulting in higher antibody levels. The addition of an aluminum adjuvant significantly enhanced the production of IgG and neutralizing antibodies, with the effect being particularly pronounced in the low-dose groups. In terms of protective effects, two weeks after the final immunization, C57/BL6 mice were challenged with wild type SFTSV. The high dose vaccine group demonstrated a significantly accelerated clearance of viral RNA in the blood and spleen. However, since C57/BL6 mice are not a lethal model for SFTSV infection, further research is required to evaluate the protective efficacy of the vaccine in lethal animal models.

### 5.3 Recombinant virus vector vaccines

Recombinant viral vector vaccines use genetically engineered viruses as carriers to deliver specific antigen genes (typically encoding key proteins of pathogens) into host cells, thereby stimulating an immune response in the body (95, 96).

#### 5.3.1 Recombinant vesicular stomatitis virus vector vaccine

Vesicular stomatitis virus (VSV) is a zoonotic arbovirus belonging to the Rhabdoviridae family. It has been developed as an attenuated viral vaccine vector capable of inducing robust neutralizing antibody responses and has demonstrated effective protection against lethal challenges (97, 98). Dong et al. cloned the human codon optimized Gn/Gc ORF from the Chinese lineage SFTSV AH12 strain into the rVSVΔG vector, enabling the virus to express SFTSV Gn/Gc on the surface of viral particles. This recombinant virus was named rVSV-SFTSV/AH12-GP (81). A single dose of rVSV-SFTSV/AH12-GP elicited highly efficient and broad-spectrum neutralizing antibodies in both immunocompetent C57BL/6 mice and IFNAR^−/−^ mice, providing complete protection against SFTSV challenge. Moreover, the protective efficacy of the candidate vaccine showed no significant differences across various routes of administration, including intraperitoneal injection, intravenous injection, subcutaneous injection, and intranasal administration. In IFNAR^−/−^ mice pre-immunized with the rVSV vector, vaccination with the candidate vaccine still provided protective immunity against SFTSV challenge, indicating that the vaccine efficacy was not compromised by prior immunization.

#### 5.3.2 Recombinant vaccinia virus vector vaccine

The LC16m8 strain of vaccinia virus (m8) and Modified Vaccinia Ankara (MVA) are classified as third-generation smallpox vaccines, characterized by high attenuation while retaining immunogenicity (99). Yoshikawa et al. utilized the highly attenuated yet immunogenic vaccinia virus strain LC16m8 (m8) as a recombinant vaccine for SFTS, expressing the SFTSV nucleoprotein (m8-N), envelope glycoprotein precursor (m8-GPC), and both N and GPC (m8-N+GPC) (82). Their m8-based SFTSV vaccines expressed SFTSV genes in infected cells, and particularly, cells infected with m8-GPC or m8-N+GPC produced virus-like particles (VLPs) in the supernatant of *in vitro* cultures. Subcutaneous administration of m8-based SFTSV vaccines at a dose of 1×10^6^ PFU twice in IFNAR^−/−^ mice successfully induced SFTSV-specific antibodies in all vaccine candidates and protected the mice from lethal challenges with 1×10^3^ or 1×10^5^ TCID50 of the SFTSV YG-1 strain. Additionally, mice pre-immunized with the Lister vaccinia strain also achieved protective immunity against SFTSV challenge after vaccination with m8-based SFTSV vaccines. Pathological analysis revealed no tissue pathological changes in mice immunized with m8-GPC or m8-N+GPC.Passive serum transfer experiments demonstrated that serum collected from mice vaccinated with m8-GPC or m8-N+GPC conferred protective immunity against lethal SFTSV challenge in naïve mice.

#### 5.3.3 Recombinant adenovirus vector vaccine

Adenoviruses are widely distributed in nature and possess high genomic manipulation flexibility, allowing the integration of large foreign gene fragments. This feature makes them an ideal choice for constructing specific vaccines (100, 101). Zhao et al. developed a recombinant replication-deficient human adenovirus type 5 (Ad5) encoding rabies virus (RABV) G and SFTSV Gn (Ad5-G-Gn) (83). Ad5-G-Gn immunization activated more dendritic cells (DCs) and B cells in lymph nodes (LNs) and induced a Th1/Th2-mediated response in splenocytes, leading to the robust production of neutralizing antibodies against SFTSV and RABV. Additionally, in 6 to 8-week-old C57/BL6 mice infected with SFTSV, Ad5-G-Gn immunization significantly reduced the SFTSV viral load in the spleen. However, this study has limitations due to the use of a non-lethal mouse model, and further safety and protective efficacy evaluations are needed.

Subsequently, the team developed Ad5 vector vaccine candidates expressing different regions of SFTSV glycoprotein (Gn, Gc, and Gn-Gc) (84). Compared to Ad5-Gc and Ad5-Gn-Gc, Ad5-Gn rapidly recruited/activated DCs, promoted B cell activation, induced specific T cells, and quickly generated high levels of SFTSV virus-neutralizing antibodies (VNA) in wild-type mice. Furthermore, in lethal SFTSV-infected IFNAR^−/−^ mice, Ad5-Gn provided complete protection and safeguarded the spleen, liver, brain, lungs, and other organs from SFTSV-related pathological changes. Their findings suggest that Gn is an advantageous target for the development of SFTSV vaccines and antibodies.

### 5.4 Subunit vaccines

Subunit vaccines, due to their excellent safety, stability, and relatively mature production techniques, have been successfully applied in the prevention of viruses such as hepatitis B virus (HBV) and human papillomavirus(HPV) (102, 103). However, they also have the issue of low immunogenicity, requiring the addition of adjuvants and multiple doses to enhance their immune effects.

#### 5.4.1 NSs recombinant protein vaccine

In the early research by Liu et al., the effectiveness of SFTSV non-structural protein (NSs) as a vaccine component was evaluated (85). However, C57BL/6 mice immunized with purified recombinant NSs combined with complete Freund’s adjuvant showed no significant difference in viremia levels compared to the control group after SFTSV challenge. Therefore, the NSs vaccine did not promote the clearance of SFTSV in mice.

#### 5.4.2 Self-assembling Gn Head-Ferritin nanoparticles vaccine

Kim et al. applied self-assembling ferritin nanoparticles fused with the head region of SFTSV Gn (GnH) to construct GnH-FT (87). The immunogenicity of the vaccine was evaluated in BALB/c mice and aged ferrets. The results showed that immunization with 1 μg of GnH-FT nanoparticles could induce a robust neutralizing antibody (NAb) response and T-cell immunity against SFTSV Gn in mice. Immunized aged ferrets not only effectively induced total IgG antibodies and NAb antibodies but also provided complete protection against SFTS symptoms and lethal SFTSV challenge.

#### 5.4.3 NP, Gn and Gc recombinant protein vaccine

Recently, Kim et al. evaluated the efficacy of recombinant protein vaccines using purified nucleocapsid protein and surface glycoproteins, assessing their immunological effects both individually and in combination (86). Immunization with either the NP or Gn subunit alone provided partial protection to IFNAR^−/−^ mice, with survival rates of 66.7% and 16.7%, respectively, while Gc vaccination failed to offer significant protection, resulting in 100% mortality (86). Among the tested recombinant protein combinations (Gn + NP, Gc + NP, and Gn + Gc + NP), the Gc + NP combination showed the highest protective effect following exposure to a lethal dose of SFTSV, achieving the highest survival rate (85.7%) and highlighting its potential as a vaccine candidate. However, NP antibodies did not exhibit neutralizing activity, and their potential role in antiviral immunity requires further investigation. All IFNAR^−/−^ mice vaccinated with single subunit vaccines succumbed to viral infection within 12 months, suggesting that a combination of protective antigens and adjuvant systems is still needed to ensure long-term humoral and cellular immunity.

### 5.5 DNA vaccines

The advantages of DNA vaccines include eliciting strong immune responses, ease of development, and the ability to quickly test multiple candidate antigen designs (104). However, they also face challenges such as the need for delivery via electroporation and insufficient immune durability, which requires multiple doses.

Kwak et al. constructed a DNA vaccine using pVax1 as an expression vector, encoding the full-length Gn, Gc, N, NS, and RNA-dependent RNA polymerase (RdRp) genes of SFTSV, based on the sequences of 31 clinical isolates from patients in China, Korea, and Japan (88). Intramuscular injection of the SFTSV DNA candidate vaccine in BALB/c mice and aged ferrets induced strong SFTSV specific T cell responses and neutralizing antibody responses. In aged ferrets (>4 years old), three intradermal immunizations of the SFTSV DNA candidate vaccine at 2-week intervals were followed by a viral challenge two weeks after the final vaccination. All vaccinated ferrets were fully protected from a lethal SFTSV challenge. However, a single dose vaccination only provided partial protection. Furthermore, their study found that Gn and Gc specific immune responses play a crucial role in preventing fatal SFTSV infection, while non-envelope specific T-cell responses also contribute to cellular protection against SFTSV infection.

Kang et al. developed another recombinant plasmid DNA (pSFTSV) as a DNA vaccine candidate, encoding the extracellular domains of Gn and Gc as well as an NP-NS fusion antigen (89). IL-12, a key factor for type 1 helper T cell (Th1) differentiation, enhances cellular immune responses. To improve protective efficacy, they incorporated IL-12 into pSFTSV, creating pSFTSV-12. The vaccine was administered to IFNAR^−/−^ mice three times via *in vivo* electroporation. Following a lethal SFTSV challenge, mice vaccinated with pSFTSV-12 showed a 100% survival rate, while those vaccinated with pSFTSV alone had only a 40% survival rate. In the presence of IL-12 expression, virus antigen specific T cell responses were significantly enhanced. However, no neutralizing antibodies were detected in the immunized mice. These data indicate that the expression of IL-12 enhances the efficacy of the vaccine, but further studies are needed to optimize the combination of appropriate target antigens and the selection of adjuvants for DNA vaccines.

### 5.6 mRNA vaccines

mRNA vaccines, with their unique advantages of high efficiency, strong adaptability, simple antigen design, short production cycle, and high safety, have provided an important means of responding to major public health emergencies (105). During the SARS-CoV-2 pandemic, Pfizer/BioNTech’s BNT162b2 and Moderna’s mRNA-1273 proved to be highly effective against SARS-CoV-2 and were developed and administered to millions of people worldwide at unprecedented speed. These two vaccines marked the first clinical approval of mRNA vaccines (106–108). The success of mRNA vaccines against SARS-CoV-2 has sparked widespread interest in the use of mRNA for the prevention and treatment of various conditions. Currently, in addition to SARS-CoV-2 vaccines, research is being conducted on various mRNA-based vaccines for infectious diseases, cancer vaccines, and mRNA-based therapeutic approaches (109–111).

Beyond the subunit vaccine platform, Kim et al. also applied the SFTSV Gn Head region (sGn-H) and SFTSV Gn Head region ferritin nanoparticles (sGn-H-FT) to the SFTSV mRNA platform (90). They encapsulated mRNA encoding sGn-H or sGn-H-FT into lipid nanoparticles (LNPs) for effective delivery. Female BALB/c mice aged 6–8 weeks were selected and intramuscularly immunized with 1 µg of sGn-H or sGn-H-FT mRNA LNPs at weeks 0 and 3. The results showed that sGn-H and sGn-H-FT mRNA LNPs exhibited strong activity, inducing effective humoral immunity in the immunized mice. By week 15 post-immunization, both total IgG and neutralizing antibodies (NAbs) remained at elevated levels. Furthermore, IFNAR^−/−^ mice immunized with sGn-H or sGn-H-FT mRNA LNPs successfully survived a lethal challenge with SFTSV. These mice experienced minor weight loss but recovered fully and rapidly. Their findings suggest that sGn-H and sGn-H-FT are promising vaccine antigen candidates capable of providing protection against SFTSV infection.

After that, Kim constructed a LNP-encapsulated mRNA vaccine expressing SFTSV Gn (91). Six-week-old C57BL/6 mice were immunized twice with LNP-encapsulated mRNA-Gn or PBS through intramuscular injection with a 14 day interval. The vaccine successfully induced robust humoral and cellular immunity in the mice. In subsequent challenge experiments, the mRNA-Gn mice showed effective protective effects.

Recently, Lu et al. developed an mRNA vaccine encoding the full-length SFTSV GP (92). The vaccine successfully induced humoral immunity and Th1-biased cellular immune responses in BALB/c mice. In IFNAR^−/−^ mice challenged with a lethal dose of SFTSV, 1 µg of the vaccine provided 100% protection, and 0.1 µg provided 90% protection. Subsequently, researchers conducted a SFTSV challenge experiment 21 weeks after vaccination at a dose of 5μg, and the mice maintained a 100% survival rate, successfully validating the long-term protective effect induced by the vaccine. Additionally, the full-length SFTSV glycoprotein mRNA vaccine also provided cross-protection against Heartland virus and Guertu virus, revealing a potential strategy for a broad-spectrum Bandavirus vaccine.

### 5.7 SFTSV vaccine targets

In summary, the development of vaccines against SFTSV currently encompasses various technological platforms, each facing challenges in selecting critical targets during the development process. Therefore, in-depth research on the key antigenic regions and immune response mechanisms of SFTSV is of great significance for optimizing vaccine design and enhancing vaccine efficacy. Gn is a core component in the processes of viral entry and membrane fusion, and it is also a major antigenic element. The domain III of its head structure has been identified as a specific target for neutralizing antibodies, while domain II may serve as an ideal binding site for broadly neutralizing antibodies (33). Identifying key antigenic regions that effectively induce neutralizing antibodies is essential for the development of certain types of vaccines.

Studies using cryo-electron microscopy (cryo-EM) on SFTSV have revealed that the aggregation of the Gn head on top of the Gc subunit forms a crown-like structure, making it less accessible to solvent. This aligns with observations that many neutralizing antibodies inhibit viral infection by targeting the Gn head, indicating that the Gn head is an ideal candidate for developing subunit vaccines. The subunit vaccine and mRNA vaccine based on GnH constructed by Kim et al. provided strong support for this finding (87, 90). The N914 glycosylation site on Gc is crucial for the assembly of viral particles and is highly conserved among bunyaviruses, making it a promising target for developing broad-spectrum protective vaccines (112).

While clarifying the key antigenic regions, the selection of standard strains is also of great importance. Researchers have found that the HB29 viral strain exhibits strong cross-reactivity with heterologous antibodies and demonstrates high neutralizing efficacy with sera from 33 SFTS patients. This indicates that the HB29 strain possesses broad immunogenicity and holds potential as an optimal standard strain for vaccine development (113). Additionally, the aforementioned live-attenuated vaccine based on the HB29 strain has shown cross-protective benefits against heterologous genotype B strains (79). mRNA vaccine candidates constructed using the HB29 strain by Lu et al. further demonstrated cross-protection against Heartland virus and Guertu virus (92).

The development of broad-spectrum vaccines against Severe Fever with Thrombocytopenia Syndrome Virus (SFTSV) represents a critical strategy to address its genetic diversity and high mutability, requiring the integration of multi-target design and advanced technological platforms. Specifically, one approach is to draw insights from broad-spectrum coronavirus vaccine development by employing a multivalent antigen combination and conserved epitope-targeting strategy (114). Incorporating antigens from different SFTSV genotypes into vaccine design could enhance the induction of broadly neutralizing antibodies. Additionally, targeting highly conserved epitopes within SFTSV, such as the N914 glycosylation site on the Gc protein, as a central antigenic determinant may help mitigate the risk of immune evasion caused by viral mutations. Second, advanced vaccine platforms should be strategically employed. mRNA vaccines offer significant advantages due to their flexibility and high efficiency, enabling rapid antigen optimization through sequence modifications. Moreover, integrating artificial intelligence to predict potential mutation sites and design antigenic compositions that cover multiple genotypes presents an innovative and forward-looking strategy for broad-spectrum vaccine development.

### 5.8 Challenge and prospect of candidate vaccines

Although various technological approaches to developing SFTSV vaccines have shown efficacy in preventing infections in animal models, limitations persist due to gaps in understanding pathogenic mechanisms and immune response pathways.

Live attenuated and inactivated vaccines, as traditional platforms, leverage well-established development systems that have significantly contributed to many viral vaccines. Live attenuated vaccines reduce pathogen virulence while preserving immunogenicity, eliciting robust immune responses and protective efficacy. However, they carry risks of mutation reversion to virulence, or excessive immune reactions. Inactivated vaccines offer greater superior safety and stability compared to live attenuated vaccines but often require multiple doses and adjuvants. Moreover, their protective efficacy in pathogenic animal models requires further evaluation. Protein subunit vaccines also excel in safety and stability, with several candidates demonstrating protective efficacy in ferrets and immunodeficient mice. However, their high production costs and reliance on adjuvants to enhance efficacy pose challenges. Recombinant vector vaccines exhibit robust protective effects in immunodeficient animals. However, their complex production process for this technology is relatively complex, and pre-existing immunity may impact vaccine efficacy. Compared with traditional vaccines, nucleic acid vaccines provide flexibility in target selection, enabling rapid testing of multiple antigen designs. Their shorter production cycles make them well-suited for addressing public health emergencies. DNA vaccine candidates, however, face challenges such as lower immunogenicity and the need for multiple doses, necessitating further research into optimization of target design and adjuvant selection. mRNA vaccines, which respond rapidly adapt to pathogen mutations, allow for prompt optimization for variants and subtypes. They also show potential for broad-spectrum antiviral effects. However, their long-term immune efficacy requires further investigation.

When comparing vaccine candidates, it is critical not only to evaluate the levels of neutralizing antibodies they elicit but also to remain vigilant for the risk of antibody-dependent enhancement (ADE) mediated by non-neutralizing antibodies. Although antibodies against the nucleocapsid protein are detectable early in infection, they lack significant neutralizing activity and may, in some cases, form sub-neutralizing antibodies that could theoretically promote viral entry into immune cells via Fcγ receptors (115). While no experimental evidence of ADE exists for SFTSV infection, experiences with dengue and other viruses suggest that non-neutralizing or low-affinity antibodies, once bound to the virus, can be internalized through specific Fcγ receptor-mediated pathways, enhancing viral replication (116). Therefore, in evaluating various vaccine platforms, it is crucial to prioritize those that induce robust neutralizing antibody responses while minimizing the generation of large quantities of non-neutralizing antibodies.

Building on a comprehensive understanding of the strengths and limitations of current vaccine platforms under investigation, future research should focus on groundbreaking strategies to advance next-generation SFTSV vaccines with improved efficacy and safety through innovations in antigen design, delivery systems, and immunization protocols. For antigen design, insights from RSV and SARS-CoV-2 vaccine development, such as stabilizing the pre-fusion conformation of viral surface proteins could be adapted. High-resolution structural characterization of neutralizing epitopes via cryo-EM could guide site-directed mutations or fusion tags to lock Gn/Gc antigens into their most immunogenic conformations, thereby enhancing the specificity and potency of neutralizing antibodies (117, 118). Delivery system optimization may incorporate novel adjuvants like TLR7/8, STING, or RIG-I agonists to boost antigen presentation and immune activation (119–121). Additionally, developing intranasal or oral vaccine formulations could induce mucosal IgA responses and localized cellular immunity in the upper respiratory tract and gut, establishing a frontline defense at viral entry sites (122).

## 6 Discussion

SFTS poses a significant global pandemic risk, threatening public health worldwide. In endemic regions, persistent tick-borne transmission of SFTSV severely endangers local populations. Consequently, research into SFTSV prevention and treatment is of paramount importance.

Significant progress has been achieved in developing animal models for SFTSV infection, yet limitations persist. Future research should prioritize establishing more effective, broadly applicable animal models to address SFTSV’s public health challenges. Integrating animal study data with clinical data from human infections will provide deeper insights into viral pathogenesis and support the development of models that accurately reflect human clinical manifestations. Additionally, models accounting for variations in age and immune status should be developed to explore how aging, comorbidities, and immunosuppression influence SFTSV infection. Such models will offer valuable insights for vaccine design and personalized therapies. Humanized mouse and non-human primate models show great promise for SFTSV research but require further optimization to enhance practicality, standardization, and reproducibility. Refining these models will improve their utility for preclinical evaluation of vaccines and antiviral therapies.

Vaccination remains the most effective strategy for preventing infectious disease outbreaks. However, progress in developing prophylactic SFTSV vaccines has been slow. All candidate vaccines are currently in preclinical stages, validated only in small animal models, with no studies in large animal models or human clinical trials. Homologous recombination in SFTSV within hosts or arthropod vectors presents a major challenge for vaccine development, contributing to immune evasion, viral diversity, enhanced virulence, and technical adaptability issues. As SFTSV is primarily endemic to certain Asian regions with low global incidence, limited market demand, prolonged development cycles, and high costs reduce commercial incentives for vaccine development. Furthermore, vaccine development faces risks that require careful evaluation, including reversion to virulence in live attenuated vaccines, interference from pre-existing immunity in viral vector vaccines, potential autoimmune reactions from mRNA vaccines, and ADE observed in dengue vaccines (123, 124). These risks underscore the need for systematic assessment in SFTSV vaccine development. Addressing these challenges demands an integrated approach combining monitoring, research, and immunization strategies to bridge basic research and practical application. Continuous monitoring of viral mutations and antigenic variations, alongside multi-target strategies and advanced technological platforms, can enhance vaccine efficacy, broaden protection against diverse viral variants, and improve immunogenicity.

In the absence of an approved vaccine, targeted public health measures are essential to reduce infection risks for high-risk groups, such as the elderly, farmers, and veterinarians. In Daishan County, Zhejiang Province, China, successful prevention and control measures have been implemented (125). These include habitat cleanup through regular removal of shrubs and fallen leaves around villages to create tick-free buffer zones, chemical tick control using long-lasting pyrethroid insecticides in farmlands, forest edges, and recreational areas, and health education campaigns via village broadcasts, pamphlets, and household visits to promote tick-bite prevention and early medical intervention. Additionally, livestock management practices, such as regular insecticidal dips for cattle and sheep, minimize tick-borne pathogen transmission. These efforts reduced the annual incidence rate in Daishan County by an average of 39.98% per year from 2015 to 2019 (APC = –39.98%, P < 0.001), maintaining low and stable incidence since 2019.

Future SFTSV research should emphasize multidisciplinary collaboration, integrating virology, immunology, epidemiology, bioinformatics, computational biology, and structural biology to create a comprehensive research framework bridging fundamental studies and clinical applications. In viral pathogenesis and immune regulation, high-throughput sequencing and genome editing technologies should be leveraged to investigate SFTSV genomic evolution, elucidate molecular mechanisms of infection, identify key pathogenic factors, and explore immune evasion strategies. A deeper understanding of host antiviral immune responses will guide vaccine design and antiviral therapies. Epidemiological studies should utilize large-scale population surveys and mathematical modeling to characterize SFTSV transmission dynamics. Geographic Information System (GIS) technologies can enable spatial-temporal analysis of viral spread, identifying high-risk zones and predicting outbreak hotspots. Efforts should also enhance vaccination feasibility and acceptance while developing targeted prevention and control measures for diverse populations. In computational biology, artificial intelligence and deep learning can analyze viral genetic variations, predict mutation sites, and identify conserved antigenic epitopes to improve broad-spectrum vaccine efficacy. In drug development, integrating structural biology and computational chemistry can optimize small-molecule antiviral drugs and design neutralizing antibodies, enhancing therapeutic outcomes. Future studies should also evaluate long-term immune responses following SFTSV infection, assessing the feasibility of convalescent plasma therapy and monoclonal antibody treatments.
